# Electrical Resistivity Response and Mesoscopic Simulation of Concrete Containing a Composite Low-Freezing-Point Powder Under Subzero Conditions

**DOI:** 10.3390/ma19214640

**Published:** 2026-10-31

**Authors:** Yangjun Deng, Xueqian Wang, Mingming Zhu, Dong Gao, Sheng Qiang

**Affiliations:** 1 College of Water Conservancy and Hydropower Engineering, Hohai University, Nanjing 210098, China; 19825120291@163.com; 2 Liaoning Provincial Water Conservancy and Hydropower Survey, Design and Research Institute Co., Ltd., Shenyang 110002, China; xueqian0108@126.com; 3 Liaoning Liaodong Peninsula Water Co., Ltd., Dalian 116000, China; mingm0510@163.com (M.Z.); gaodong_cheerful@163.com (D.G.)

**Keywords:** composite low-freezing-point powder, electrical resistivity, compressive strength, mesoscopic current-field model

## Abstract

Abrupt temperature drops during cold waves pose a threat to early-age concrete in cold regions. This study combined electrical resistivity monitoring, compressive strength testing, and mesoscopic electrical modeling to investigate the performance of concrete containing a composite low-freezing-point powder (CLFP). CLFP comprised calcium nitrate tetrahydrate and sodium nitrite at a mass ratio of 3:1. Five dosages of 0%, 2%, 4%, 6%, and 8% were investigated, and the specimens were cured at −20 °C for 3 d before being transferred to 20 °C. A random-aggregate model was used to link experimentally measured time-dependent mortar conductivity to the overall electrical resistivity of concrete, and the electrical responses were evaluated alongside subsequent strength development. All mixtures exhibited three resistivity stages: slow increase, rapid rise, and stabilization. Increasing the CLFP dosage delayed the resistivity-derived apparent freezing onset by up to 0.43 h and reduced the stabilized resistivity by 29.96%. After 28 d of subsequent curing at 20 °C, the 2% CLFP mixture achieved the highest compressive strength of 37.6 MPa, approximately 7.1% higher than the reference value of 35.1 MPa. The 4%CLFP mixture reached 35.4 MPa, whereas the 6% and 8% mixtures reached only 24.1 MPa and 25.3 MPa, respectively. The model reproduced the decrease in stabilized resistivity with increasing CLFP dosage, with relative errors of 1.5–8.4% for the 0–6%CLFP mixtures and 18.2% for the 8% mixture. Considering both delayed apparent freezing onset and strength retention, 4%CLFP was identified as a suitable dosage for balancing these two indicators under the tested conditions.

## 1. Introduction

Concrete on dam surfaces in cold regions is exposed to subzero temperatures, cold waves, and freeze–thaw cycles over extended periods. Freezing and repeated phase changes in near-surface pore water can generate frost-heave pressure, causing interfacial weakening, microcrack propagation, and reduced resistance to water penetration, thereby compromising structural durability and operational safety. During construction and early-age curing, the relatively low degree of cement hydration makes concrete particularly susceptible to irreversible damage from sudden exposure to subzero temperatures. Cai et al. [1] identified pore-water freezing as a key process governing the initiation and development of freeze–thaw damage in concrete. Ge et al. [2] demonstrated that rapid cooling causes freezing-induced stress to develop earlier and increase substantially, promoting cumulative microcrack propagation. Hu et al. [3] found that subsequent deterioration caused by early-age freezing is closely related to the onset, temperature, and duration of freezing. In freeze–thaw tests on pervious concrete, Guo et al. [4] observed that higher initial porosity accelerates pore-structure deterioration and strength loss, further highlighting the importance of internal pore structure in frost damage development. Therefore, delaying extensive pore-water freezing and mitigating early-age frost damage are important objectives in protecting concrete on dam surfaces in cold regions.

Previous research on early-age frost damage has examined antifreeze materials and curing regimes. Wu et al. [5] identified the incorporation of antifreeze components and appropriate precuring as effective approaches to improving concrete performance at subzero temperatures. Inorganic salts, such as nitrates and nitrites, can lower the freezing point by altering the pore-solution composition while also influencing cement hydration and strength development. Karagöl et al. [6] found that calcium nitrate improves the compressive strength of concrete exposed to low temperatures. Kothari et al. [7] investigated the effects of nitrate salts and concrete composition on pore-water phase transitions at subzero temperatures. Alzaza et al. [8] demonstrated that combining chloride-free antifreeze components, calcium silicate hydrate (C–S–H) seeds, and room-temperature precuring improves hydration and strength development in cement paste cured at low temperatures. Regarding composite antifreeze admixtures, Karagol et al. [9] and Khan et al. [10] investigated calcium nitrate–urea and calcium nitrate–sodium thiocyanate systems, respectively, and found that combining these components improves strength under specific low-temperature curing conditions. These studies provide a basis for designing composite antifreeze materials; however, the effectiveness of different combinations requires verification for the relevant material compositions and curing conditions.

The freezing-point depression provided by antifreeze components does not necessarily correspond to their effects on setting acceleration and early-age strength development. Choi et al. [11] found that increasing the dosage of a calcium nitrite–calcium nitrate accelerator enhances early-age strength but may adversely affect later-age strength. Yoneyama et al. [12] further demonstrated that this type of accelerator promotes early hydration and the formation of nitrite/nitrate-AFm phases, with the resulting hydration products contributing to pore filling. Zhang et al. [13] found that sodium nitrite produces substantial freezing-point depression in a blended cement system at −10 °C, whereas excessive dosages may inhibit hydration. Choi et al. [14] developed a maturity equation for subzero temperatures that accounts for antifreeze admixtures, demonstrating that strength development also depends on the temperature–time history. Consequently, the dosage of composite low-freezing-point powder should be evaluated by considering both its ability to delay freezing and its effects on strength development after freezing, rather than relying solely on freezing-point depression or early-age strength enhancement.

Deformation monitoring, liquid-water characterization, and electrical measurements have been used to identify early-age freezing and the associated damage. Zhang et al. [15] found that frost-heave deformation and residual deformation can indicate the extent of frost heave and frost damage in early-age concrete, respectively. Li et al. [16] used low-field nuclear magnetic resonance to monitor the distribution and evolution of liquid water in situ at subzero temperatures. Under their experimental conditions, the antifreeze admixture primarily mitigated frost-heave damage, while its effect on promoting hydration at subzero temperatures was limited. The electrical resistivity of concrete is closely related to the ionic concentration of the pore solution, liquid-phase connectivity, and pore structure. Pore-water freezing reduces conductive liquid pathways, resulting in a marked increase in resistivity. Sang et al. [17] assessed the freezing process of early-age concrete using electrical resistivity measurements, while He et al. [18] established relationships between electrical resistivity, porosity, and pore-network connectivity. These studies support the use of electrical resistivity to track freezing. However, low-freezing-point powder alters both the ionic concentration of the pore solution and the freezing state. Its electrical response therefore needs to be interpreted in conjunction with strength measurements and an analysis of the constituent phases.

Mesoscopic numerical simulations provide a means of examining electrical conduction within concrete at the scale of the mortar matrix, aggregates, and interfacial transition zones. Li et al. [19] investigated the effects of coarse aggregates and interfacial transition zones on the electrical resistivity of conductive concrete. The study by Chai et al. [20] on the freezing behavior of ionic solutions within gel pores provided insights into the phase transitions of salt solutions under confinement. Nevertheless, for concrete containing a composite low-freezing-point powder comprising calcium nitrate tetrahydrate and sodium nitrite, the relationship between the dosage-dependent, time-varying electrical properties of the mortar phase and the overall resistivity response of concrete under subzero conditions remains insufficiently established. Existing constituent-phase analyses provide a basis for understanding electrical conduction, but further investigation is needed to determine how the evolving mortar conductivity translates into the rising and stabilized stages of concrete resistivity. It also remains necessary to establish whether delaying the resistivity-derived apparent freezing onset is accompanied by favorable strength development during subsequent above-zero curing.

Accordingly, this study combines electrical resistivity monitoring, compressive strength testing, and random-aggregate mesoscopic electrical modeling to investigate concrete containing different CLFP dosages. The methodological contribution lies in incorporating experimentally measured, dosage-specific mortar conductivity functions as time-dependent material inputs to calculate concrete resistivity, rather than directly fitting the concrete resistivity curves. This approach links mortar-scale electrical measurements to the overall concrete response and enables analysis of aggregate-induced current redistribution and the sensitivity of equivalent resistivity to the interfacial transition zone. Comparisons with measured concrete resistivity evaluate model agreement during both the rising and stabilized stages. By integrating these electrical analyses with compressive strength measurements after transfer to above-zero curing, the study evaluates the balance between delayed apparent freezing onset and strength retention, providing a basis for CLFP dosage selection under the tested conditions.

## 2. Materials and Methods

### 2.1. Raw Materials and Specimen Preparation

**Figure 1:**
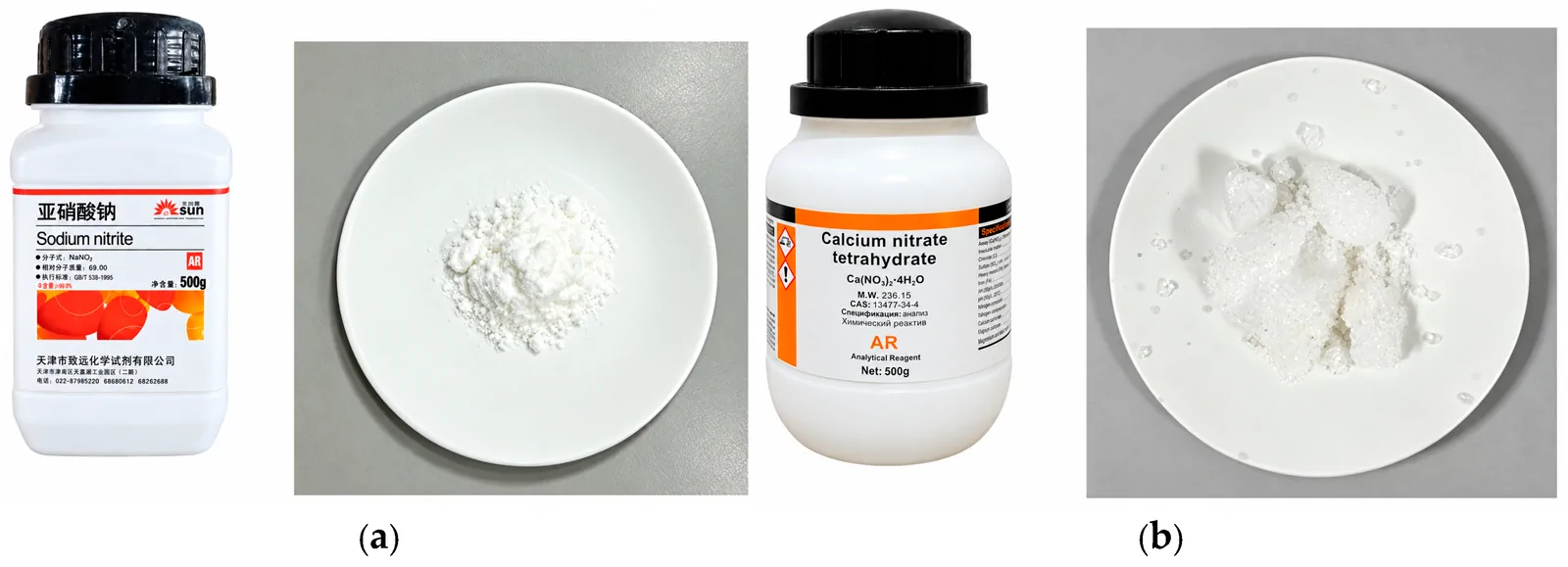
Appearance of the CLFP components: (**a**) sodium nitrite; (**b**) calcium nitrate tetrahydrate.

Ordinary Portland cement of grade P·O 42.5 was used in this study. The fine aggregate was medium sand conforming to grading Zone II, with a fineness modulus of 2.8, and the coarse aggregate had particle sizes ranging from 5 to 10 mm. A high-range water-reducing admixture was also used. The composite low-freezing-point powder (CLFP) consisted of calcium nitrate tetrahydrate and sodium nitrite, supplied by Xilong Scientific Co., Ltd. (Shantou, China) and Tianjin Zhiyuan Chemical Reagent Co., Ltd. (Tianjin, China), respectively. Karagöl et al. [6] used calcium nitrate tetrahydrate at 6% by cement mass, whereas Yin et al. [21] selected sodium nitrite at 2% by cement mass under their experimental conditions. Related patent literature disclosed nitrate-to-nitrite mass ratios ranging from approximately 1:3 to 3:1, corresponding to nitrite accounting for 25–75% of the combined salt mass. With reference to these studies, a 3:1 mass ratio of calcium nitrate tetrahydrate to sodium nitrite was adopted as a fixed experimental formulation. The component ratio was maintained while only the total CLFP dosage was varied. The detailed mix proportions of all concrete mixtures are presented in Table 1. The principal physicochemical properties and appearances of the CLFP components are presented in Table 2 and Figure 1, respectively.

The CLFP was prepared by mixing calcium nitrate tetrahydrate and sodium nitrite at a mass ratio of 3:1 and was incorporated as an addition rather than as a cement replacement. All mixtures had the same water-to-cement ratio of 0.48. The water used to dissolve CLFP was included in the total mixing water content of 240 kg/m^3^. Five concrete mixtures, designated A0–A4, were prepared with CLFP dosages of 0%, 2%, 4%, 6%, and 8% by cement mass, respectively. Five corresponding mortar mixtures, designated B0–B4, were prepared using the same CLFP dosages. A0 and B0 served as the reference concrete and mortar mixtures, respectively.

**Figure 2:**
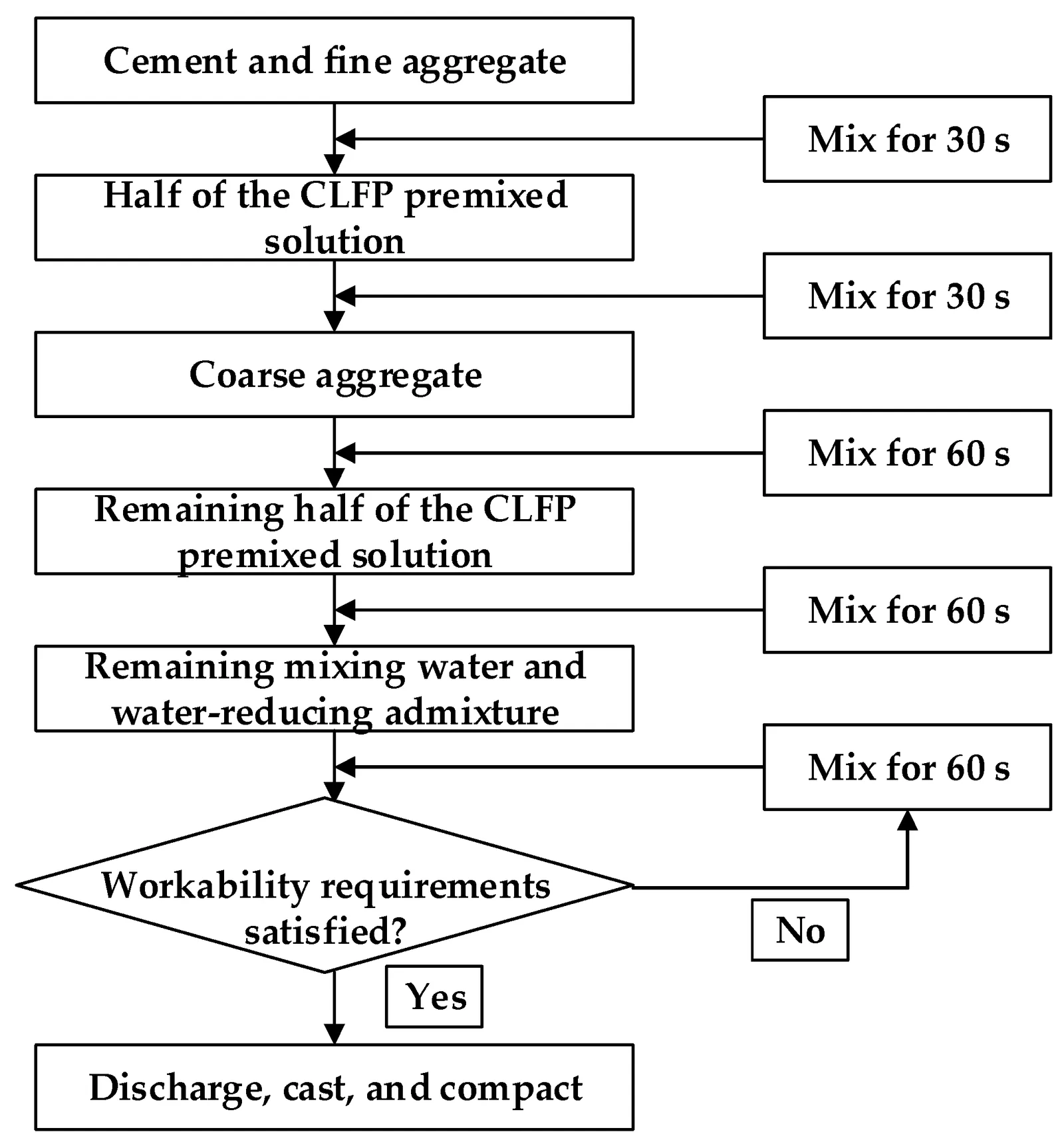
Specimen preparation procedure.

The specimens were prepared using a staged material addition procedure, as illustrated in Figure 2. After mixing, the mixtures were cast into 100 mm × 100 mm × 100 mm molds and compacted by vibration. The specimens were cured at 20 ± 1 °C and a relative humidity of at least 90% for 24 h before demolding. Tests at the designated ages were conducted in accordance with GB/T 50081-2019, Standard for Test Methods of Concrete Physical and Mechanical Properties [22].

### 2.2. Experimental Equipment and Test Methods

The main experimental equipment comprised a temperature- and humidity-controlled chamber (Suzhou Aobeisi Environmental Test Equipment Co., Ltd., Suzhou, China), a GDM-8255 dual-display digital multimeter (Good Will Instrument Co., Ltd., New Taipei City, Taiwan), and an adjustable DC power supply supplied by Guangzhou Junxiang Automation Control Equipment Co., Ltd. (Guangzhou, China).The chamber provided the subzero and above-zero curing environments. The multimeter was used to measure specimen voltage and resistance, while the DC power supply delivered a constant current to the outer electrodes during four-electrode measurements on the mortar specimens.

**Figure 3:**
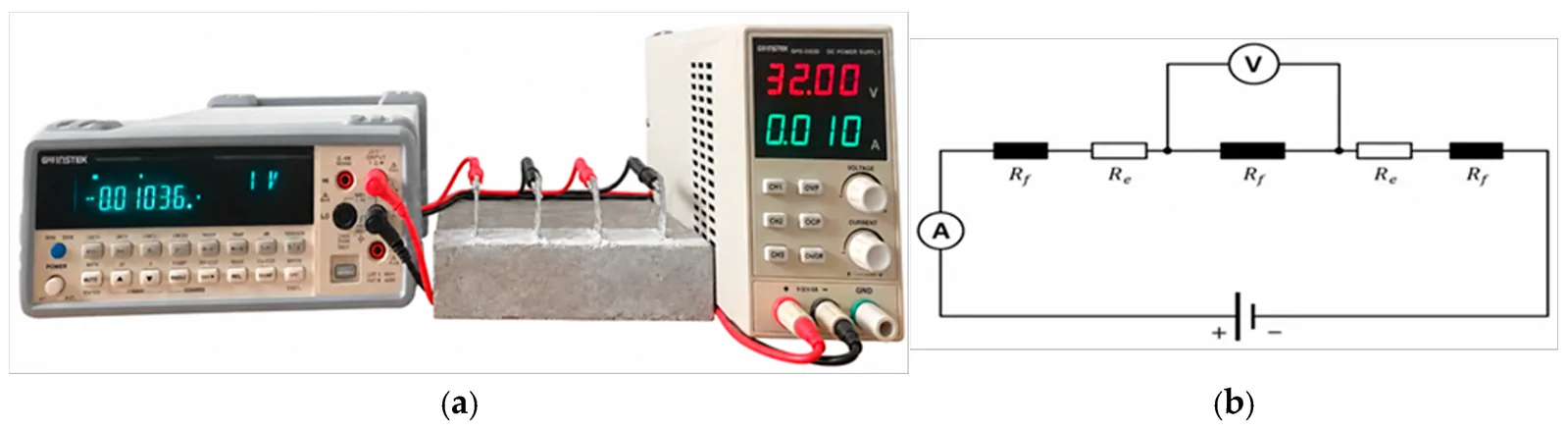
Electrical resistivity measurement: (**a**) experimental setup; (**b**) equivalent circuit.

Concrete resistivity was monitored at 20 min intervals using two embedded electrodes. This arrangement provides simple wiring and maintains the electrode contact area and measurement configuration throughout curing, allowing changes in resistivity to be tracked over time. Mortar resistivity was used to determine the electrical conductivity of the mortar matrix in the numerical model. Therefore, four embedded electrodes were used to reduce the effects of lead resistance and electrode–specimen contact resistance by separating the current and voltage electrodes. The electrodes were made of 304 stainless steel mesh with a mesh opening of 6 mm and a wire diameter of 0.6 mm, and the effective measurement spacing was 100 mm. Measurements were performed using a GW Instek GDM-8255 dual-display digital multimeter, as shown in Figure 3. For the two-electrode measurements on concrete, the test current was 100 nA, and each reading was recorded after it had remained stable for 2 s. The same electrode material, installation procedure, connection polarity, and reading procedure were used for all specimens to improve measurement consistency. Nevertheless, the two-electrode method cannot completely eliminate interfacial impedance, including contact resistance and electrode polarization. These effects were therefore recognized as limitations when comparing resistivity variations among mixtures. Three replicate specimens were tested for each group, and the results were averaged. For the 28d compressive strength, the sample standard deviation (SD) and coefficient of variation (CV) were calculated to quantify within-mixture variability. CV was calculated as SD divided by the mean and expressed as a percentage. No statistical significance tests were performed to assess differences among mixtures.

Concrete resistivity was calculated using Equation (1), and characteristic parameters, including the freezing onset time, freezing stabilization time, and stabilized resistivity, were extracted from the resistivity–time curves. Following the curvature-based method proposed by Sang [23], time was measured from the moment the specimens were placed in the −20 °C environment. The apparent freezing onset time was defined as the time corresponding to the local maximum in absolute curvature within the transition region from a slow increase to a rapid rise in the resistivity curve. The same curve-processing and curvature-calculation procedures were applied to all mixtures.

(1) $$R=\frac{U}{I},\;\rho =\frac{\mathrm{RS}}{L}$$

where *R* is the electrical resistance of the measured region (Ω), *ρ* is the electrical resistivity of concrete (Ω·m), *U* is the voltage across the measured region (V), *I* is the applied current (A), *S* is the cross-sectional area of the specimen (m^2^), and *L* is the length of the measured region (m).

**Figure 4:**
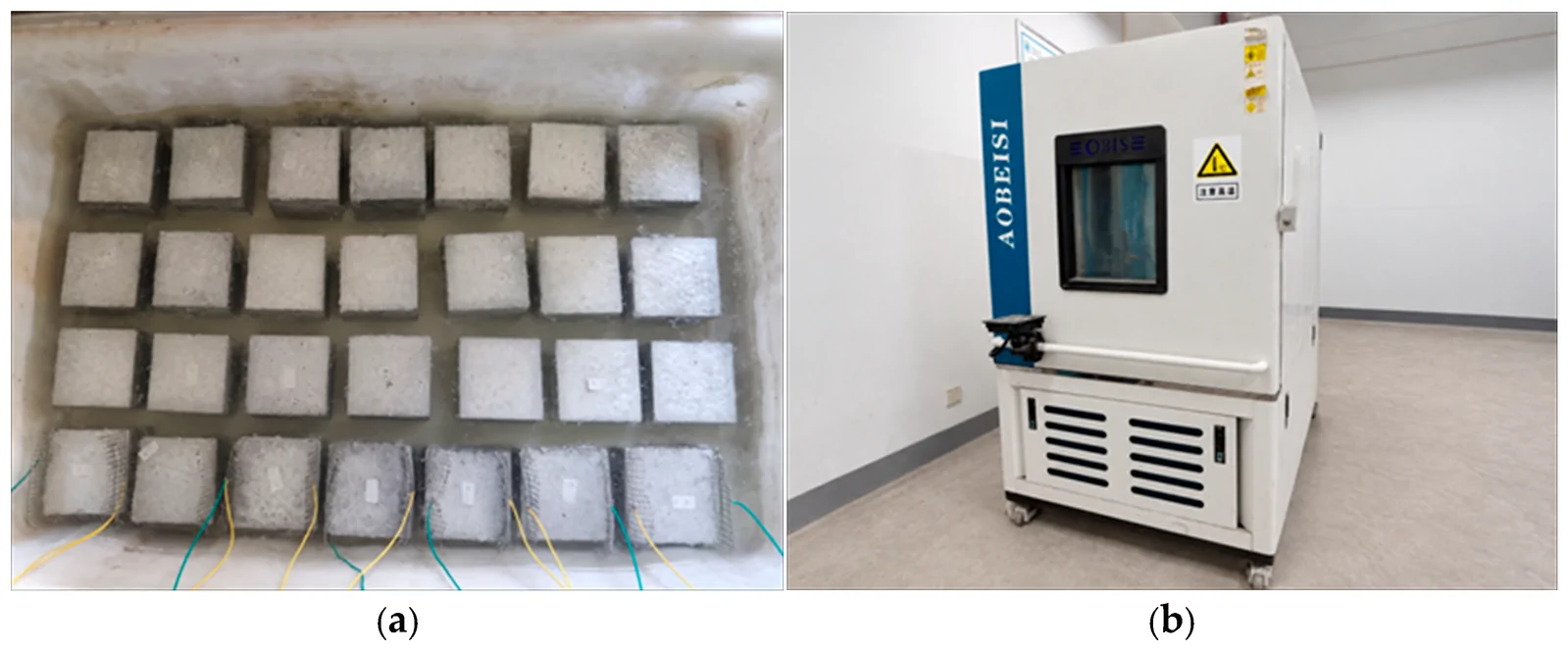
Specimen immersion and curing equipment: (**a**) specimen immersion; (**b**) temperature- and humidity-controlled chamber.

To ensure that all concrete specimens had similar initial moisture conditions, the demolded specimens were arranged by group and immersed in water in a curing tank for 24 h. During immersion, the electrode plates and wires of the instrumented specimens were kept exposed for subsequent connection to the testing equipment, as shown in Figure 4a. After immersion, the A0–A4 specimens were placed in a constant-temperature and humidity chamber and subjected to the prescribed curing regime. The specimens were first continuously cured at −20 °C for 3 d, during which their actual internal temperatures were measured using a temperature probe connected to a multimeter. They were then transferred to a 20 °C environment for further curing. Electrical resistivity and compressive strength were measured during the subzero-curing stage and after 3 d, 7 d, and 28 d of subsequent curing at 20 °C. The constant-temperature and humidity chamber is shown in Figure 4b.

### 2.3. Numerical Modeling

At the mesoscopic scale, concrete can be represented as a three-phase composite consisting of a mortar matrix, coarse aggregates, and interfacial transition zones (ITZs) [24]. In this study, a coupled discontinuum–continuum approach was adopted to generate mesoscopic concrete models by randomly placing aggregates within a prescribed domain. The computational domain measured 100 mm × 100 mm × 100 mm. The coarse aggregate particle sizes ranged from 5 to 10 mm, consistent with those used in the experiments, and the aggregate volume fraction was set to 34.4% based on the mixture proportions.

Chen et al. [25] investigated the microstructure of the concrete ITZ using environmental scanning electron microscopy. Images of the aggregate–mortar interface were divided into successive strips and binarized over a region extending approximately 48 μm from the aggregate surface. Their results indicated that ITZ porosity increased from the mortar side toward the aggregate surface, with a two-dimensional pore area fraction of 23.81% near the aggregate. This value was rounded to 24% in the present study to simplify the model. Following the approach of Li et al. [19], a two-dimensional ITZ pore model measuring 50 μm × 50 μm was constructed. Circular pores were randomly generated within the ITZ domain until their total area reached 24% of the model area, producing a two-dimensional mesoscopic model consisting of pores and the surrounding mortar matrix.

The Electric Currents interface in the AC/DC Module of COMSOL Multiphysics 6.3 (COMSOL AB, Stockholm, Sweden) was used to calculate the electric potential and current density within the model. The corresponding electrical conductivity was assigned to each material domain, and current conduction was governed by Ohm’s law, as expressed in Equation (2). Electrically insulating boundary conditions were imposed on all non-electrode surfaces, as shown in Equation (3). The two opposing electrode boundaries were assigned a ground condition and a constant-current condition, respectively, as given in Equations (4) and (5). The applied current was consistent with the experimental conditions. After solving for the electric potential and current density, the effective electrical resistivity of concrete was calculated from the potential difference between the electrodes, the applied current, and the model dimensions. The ITZ was explicitly included in the initial model, and a subsequent sensitivity analysis was performed to determine whether it needed to be represented as a separate phase in the three-dimensional model.

**Figure 5:**
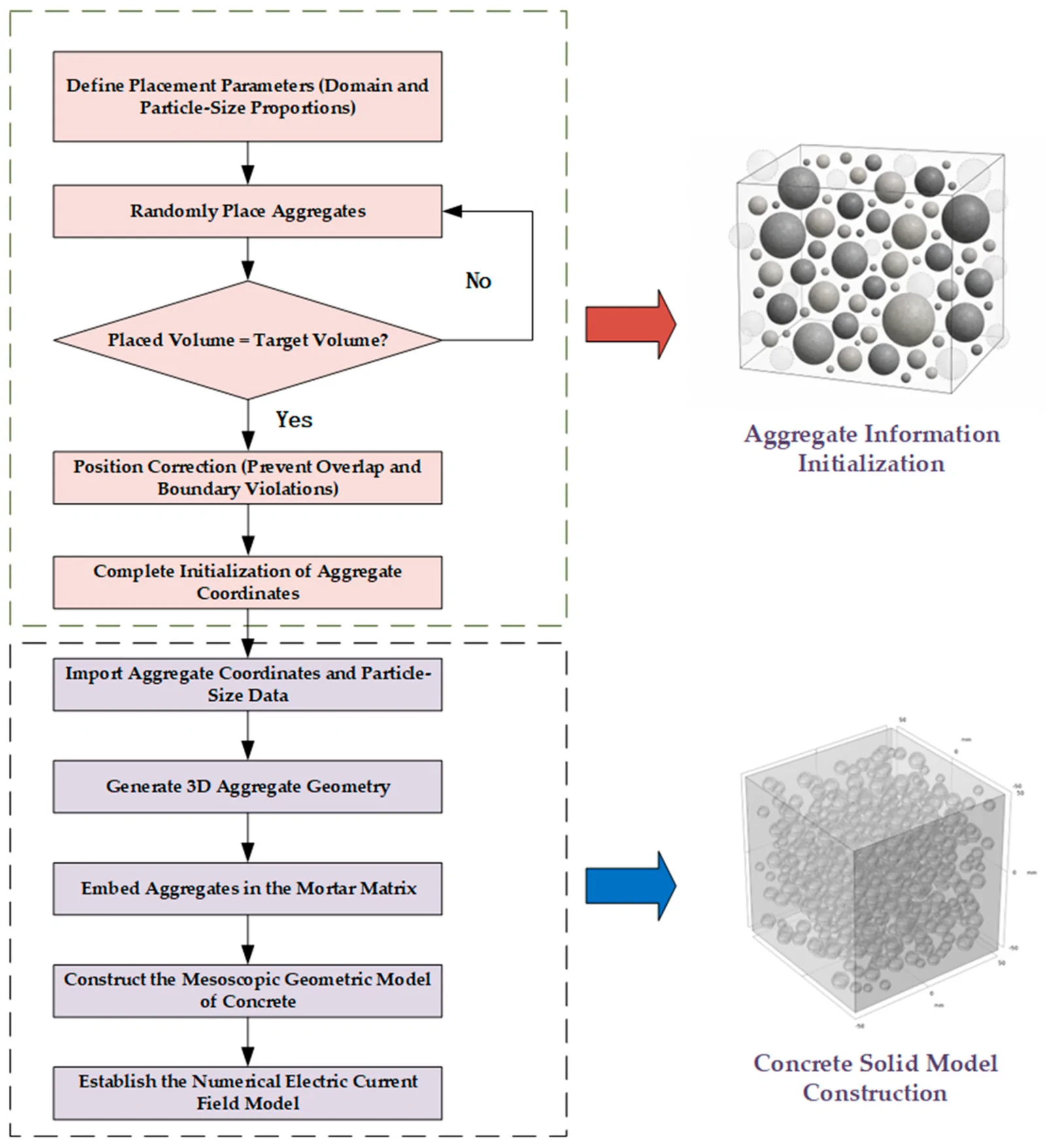
Procedure for constructing the random-aggregate concrete model.

The modeling procedure is illustrated in Figure 5.

*J* = *σ* × *E* (2)

*n* × *J* = 0 (3)

*V*_0_ = 0 (4)

(5) $$\int J\times \mathrm{nds}=I_{0}$$

where *J* is the current density (A/m^2^), *σ* is the electrical conductivity (S/m), *E* is the electric field strength (V/m), *n* is the surface normal, *V*_0_ is the initial electric potential (V), and *I*_0_ is the initial current (A).

The model parameters included the electrical resistivities of the mortar matrix, aggregates, and ITZ. The mortar matrix constitutes the principal conductive phase in concrete, and its resistivity varies with the duration of subzero exposure and the CLFP dosage. To determine the electrical properties of the mortar matrix at different dosages, resistivity measurements were performed on mortar specimens B0–B4 at −20 °C. Functions were fitted to the measured resistivity–time curves, and the fitted resistivity functions were converted into conductivity functions for use as mortar-phase material inputs in COMSOL.

The fitted functions describe the electrical properties of the mortar phase rather than those of concrete as a whole. The mesoscopic model incorporates aggregate geometry and its obstruction of current flow to calculate the effective concrete resistivity from the mortar conductivity inputs. The purpose of this representation is to resolve the spatial heterogeneity of electrical conduction, rather than to simulate freezing-induced mechanical deterioration. The measured mortar conductivity reflects the combined electrical response under the experimental conditions, but incorporating this input does not constitute thermo-mechanical or damage coupling. The model does not explicitly simulate pore-water phase changes, freezing-induced stresses, or damage evolution. Its geometry remains fixed, and crack-induced changes in conduction paths are not explicitly represented. The model therefore predicts the freezing-related electrical response rather than directly determining frost damage or strength retention. Its predictive capability is conditional on the prescribed mortar conductivity functions and model parameters. For an untested CLFP dosage or curing condition, the corresponding mortar conductivity function must first be established.

The aggregates were treated as a phase with low electrical conductivity, and their resistivity was selected with reference to natural rock materials. Common aggregates, including limestone, dolomite, and granite, generally have substantially higher resistivities than the mortar matrix, typically ranging from 100 to 2 × 10^5^ Ω·m [19]. An aggregate resistivity of 20,000 Ω·m was adopted in this study. The ITZ is a relatively weak region between the aggregate and mortar matrix, with a thickness of approximately 20–60 μm, which is much smaller than the aggregate size. It is characterized by a relatively loose microstructure, high permeability, a high degree of hydration, low strength, and susceptibility to cracking [26]. The pores were assigned the electrical resistivity of air, while the surrounding matrix was assigned the experimentally measured resistivity of the corresponding mortar mixture. The effective ITZ resistivity was calculated using the same governing equations and boundary conditions as those applied to the concrete resistivity model and was subsequently assigned to the ITZ domains in the three-phase mesoscopic concrete model.

A mesh refinement study was performed on the three-dimensional mortar–aggregate model using the A0 mortar conductivity function. Three free tetrahedral meshes containing 292,476, 626,366, and 1,224,191 domain elements were compared while keeping the geometry, material properties, and boundary conditions unchanged. For the mesh containing 626,366 elements, the maximum and minimum element size settings were 15 mm and 2.8 mm, respectively. The maximum element growth rate, curvature factor, and resolution of narrow regions were 1.6, 0.7, and 0.4, respectively. Effective resistivity was evaluated at 0, 0.10, 0.20, 0.35, and 3 d. A relative difference below 1% between successive meshes at all evaluated times was adopted as the convergence criterion. Refinement from 626,366 to 1,224,191 elements changed the calculated resistivity by 0.292–0.395%, satisfying this criterion for the overall effective resistivity of the evaluated case.

## 3. Results and Discussion

### 3.1. Effect of CLFP Dosage on the Electrical Resistivity of Concrete at Subzero Temperature

**Figure 6:**
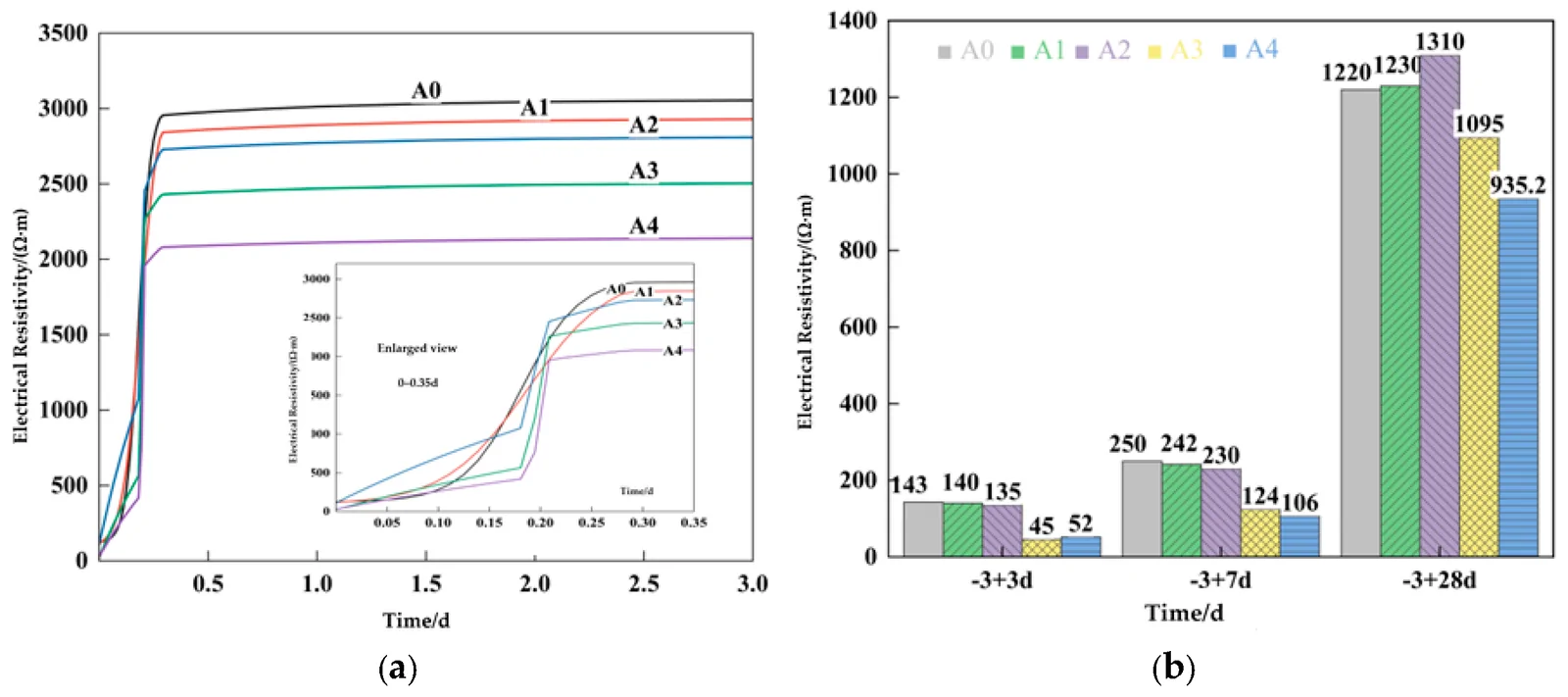
Effect of CLFP dosage on the electrical resistivity of concrete: (**a**) resistivity curves during subzero curing; (**b**) resistivity at different ages after transfer from subzero to above-zero curing.

Electrical resistivity tests were conducted on concrete specimens containing different CLFP dosages at −20 °C. After 3 d of subzero curing, the specimens were transferred to an environment maintained at 20 °C for subsequent curing. Electrical resistivity was measured after 3 d, 7 d, and 28 d of above-zero curing to evaluate the recovery of the concrete after freezing. The results are shown in Figure 6. The characteristic values obtained during the subzero temperature test are presented in Table 3.

As shown in Figure 6a, the main changes in the electrical resistivity of all concrete mixtures during 3 d of continuous curing at −20 °C occurred within the first 0.35 d. The enlarged view shows that the resistivity of each mixture underwent a slow increase, a rapid rise, and subsequent stabilization within 0.35 d. Thereafter, the resistivity increased only slightly and remained nearly stable until the end of the 3 d subzero-curing period. As the CLFP dosage increased, the freezing onset time was delayed from 0.180 d for A0 to 0.198 d for A4, while the stabilized resistivity of A4 was 29.96% lower than that of A0. Dissolution of CLFP increased the ionic concentration of the pore solution and reduced the chemical potential and freezing point of water, allowing more pore water to remain liquid at subzero temperature and thereby delaying extensive freezing [20,27]. Unfrozen water films and the mobile ions within them maintained electrically conductive pathways; consequently, a higher CLFP dosage resulted in a lower resistivity during the stabilized freezing stage.

In Table 3, the resistivity stabilization time did not vary monotonically with CLFP dosage. The duration of the rapid resistivity-rise stage, calculated as the difference between the resistivity stabilization time and the apparent freezing onset time, was 0.083 d, 0.105 d, 0.069 d, 0.023 d, and 0.014 d for A0–A4, respectively. This stage lasted slightly longer in A1 than in A0 but became shorter at CLFP dosages of 4% or higher. The fitted characteristic time describes the timescale of the resistivity transition: a smaller value indicates a more abrupt rise, whereas a larger value indicates a more gradual change. The fitted characteristic times of A0–A4 were 0.028 d, 0.036 d, 0.024 d, 0.008 d, and 0.005 d, respectively, showing a trend consistent with the duration of the rapid-rise stage. A1 exhibited the largest fitted characteristic time, indicating a relatively gradual resistivity transition. In contrast, A3 and A4 exhibited smaller fitted characteristic times, indicating that their rapid resistivity-rise stages began later but occurred over shorter periods. In addition, the initial resistivities of A3 and A4 were substantially lower than those of the other mixtures, indicating that high CLFP dosages altered the initial electrical conduction state of concrete. The apparent freezing onset time, resistivity stabilization time, and fitted characteristic time should be considered together when comparing the effects of different CLFP dosages. The apparent freezing onset time identified using the curvature criterion marks the transition to the rapid resistivity-rise stage and is associated with pore blockage caused by ice crystal growth. However, electrical resistivity is also influenced by temperature, pore solution ion concentration, and cement hydration. Accordingly, the apparent freezing onset time is used only as a comparative indicator of the freezing-delay effects of different CLFP dosages under the same experimental conditions.

As shown in Figure 6b, after the specimens were transferred to the 20 °C environment and cured for 3 d, the resistivity of every mixture decreased by more than 95% relative to that recorded during the stabilized freezing stage. After 28 d of above-zero curing, the resistivity of A0–A2 increased to 1220–1310 Ω·m, whereas that of A4 was only 935.2 Ω·m. During the initial stage of above-zero curing, melting of pore ice restored the connectivity of the liquid conductive pathways, while the increase in temperature enhanced ion mobility, resulting in a rapid decrease in resistivity. As cement hydration continued, free water was consumed and the resulting C–S–H gel gradually filled and partitioned the capillary pores. The number of conductive pathways therefore decreased, causing the resistivity to increase with curing age. The relatively low resistivity of A4 indicates that the enhanced electrical conduction associated with its higher ionic concentration partially offset the increase in resistivity caused by pore-structure densification.

### 3.2. Effect of CLFP Dosage on the Compressive Strength of Concrete After Subzero Curing

**Figure 7:**
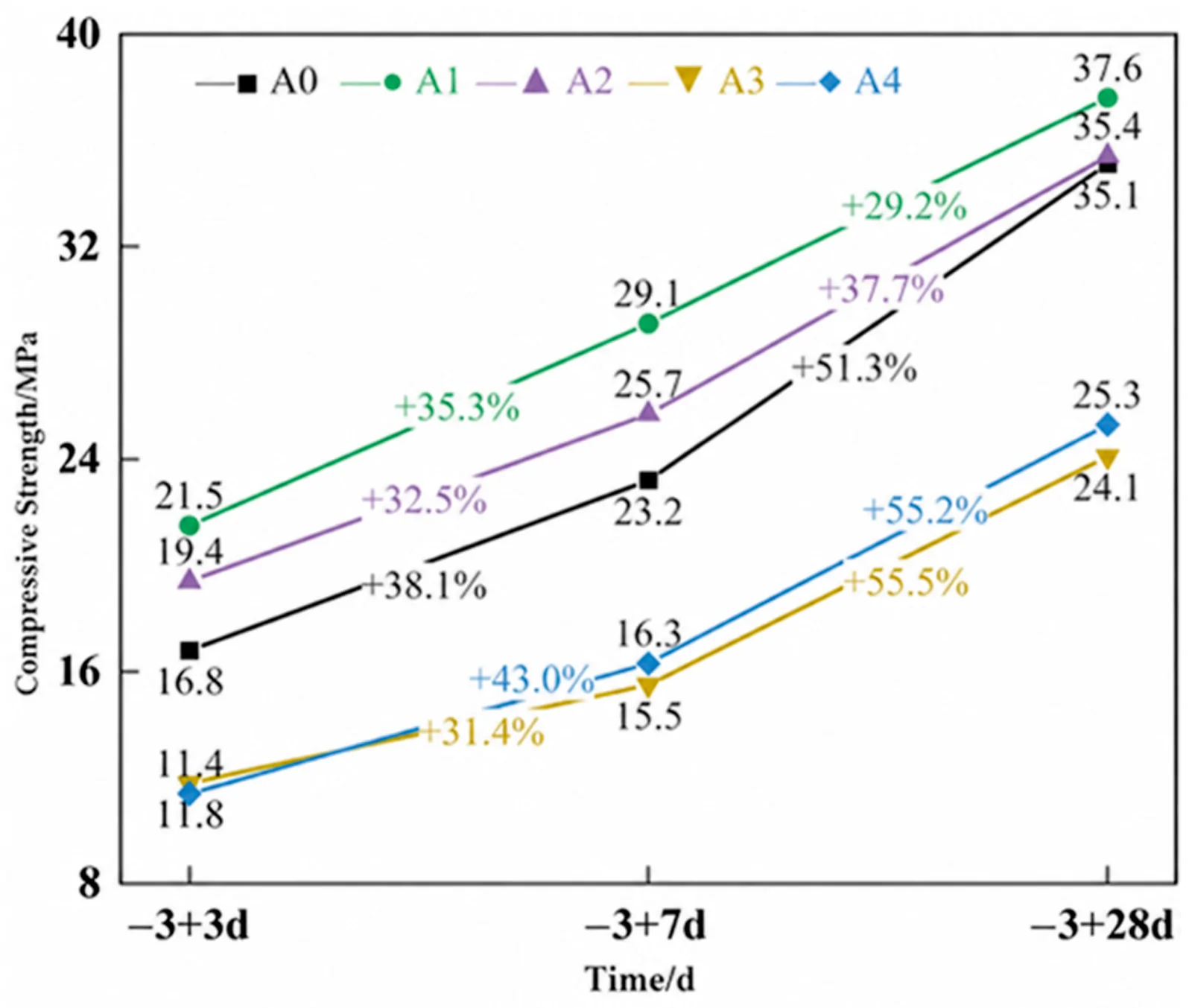
Compressive strength at different ages during above-zero curing following subzero exposure.

To evaluate the effects of CLFP dosage on frost damage and the recovery of mechanical properties during subsequent above-zero curing, the cubic compressive strength was measured after 3 d, 7 d, and 28 d of curing at 20 °C. The effects of CLFP on early-age frost damage and subsequent strength development were assessed by comparing the strength and strength gain of the different mixtures at each curing age. The results are shown in Figure 7.

As shown in Figure 7, the 28 d compressive strength of A1 reached 37.6 MPa, approximately 7.1% higher than that of A0. The strength of A2 was 35.4 MPa, which was comparable to the 35.1 MPa measured for A0. In contrast, the strengths of A3 and A4 were only 24.1 MPa and 25.3 MPa, corresponding to reductions of 31.3% and 27.9%, respectively. As shown in Table 4, the standard deviations of the 28 d compressive strength ranged from 0.361 MPa to 0.608 MPa, and the coefficients of variation ranged from 1.03% to 1.72%, indicating low variability among the three replicate specimens within each mixture. Comparisons among mixtures reflect differences in measured means rather than statistically confirmed significant differences.

This non-monotonic response indicates that increasing the CLFP dosage did not continuously improve strength. At moderate dosages, freezing-point depression can help maintain liquid water for hydration during subzero exposure, while the dissolved salts can alter early hydration reactions. Kim et al. [28] reported that appropriate dosages of nitrite-containing admixtures promoted hydration and strength development, although the benefits depended on dosage and curing age. These findings support interpreting the favorable performance of A1 and A2 in terms of the combined effects of antifreezing action and hydration development.

At higher dosages, strength development depends not only on the availability of unfrozen water but also on the hydrate assemblage, pore structure, and development of internal defects. Wan et al. [29] observed that excessive calcium nitrite addition to early-frozen cement paste was associated with increased nitrite–AFm formation, a loose microstructure, and reduced strength. Tomita et al. [30] reported increased shrinkage and cracking tendency with increasing nitrite–nitrate accelerator dosage. These studies indicate that accelerated hydration and increased salt addition do not necessarily produce a stronger load-bearing structure. Wang et al. [31] also linked the mechanical properties of early-age frozen concrete to its pore structure. Accordingly, the lower strengths of A3 and A4 should be discussed in terms of changes in hydration products, pore structure, and shrinkage-related defects, in addition to freezing damage. However, because the admixture compositions and curing conditions differed among these studies, their dosage thresholds and specific mechanisms cannot be directly transferred to the present CLFP system.

The discussion of hydration and microstructural mechanisms above is based on published studies and has not been directly verified in this study. Among the mixtures tested, the 2%CLFP mixture achieved the highest measured compressive strength, whereas the 8%CLFP mixture exhibited the latest resistivity-derived apparent freezing onset time. The 4%CLFP mixture maintained compressive strength close to that of the reference mixture while also exhibiting a later apparent freezing onset. Based on these two measured indicators, 4%CLFP was considered a suitable dosage for balancing strength retention and delayed apparent freezing onset under the present experimental conditions.

### 3.3. Effect of the Interfacial Transition Zone on the Electrical Resistivity of CLFP-Containing Concrete

The interfacial transition zone (ITZ) is a relatively weak region between the aggregate and mortar matrix. Its porosity and microstructural characteristics differ from those of the mortar matrix and may affect current-conduction pathways within concrete [32]. Song et al. [33] and Li et al. [34] demonstrated the effects of interfacial structure and ITZ thickness in recycled concrete from the perspectives of freeze–thaw resistance and chloride transport, respectively. It was therefore necessary to evaluate the influence of the ITZ on the electrical response considered in this study.

**Figure 8:**
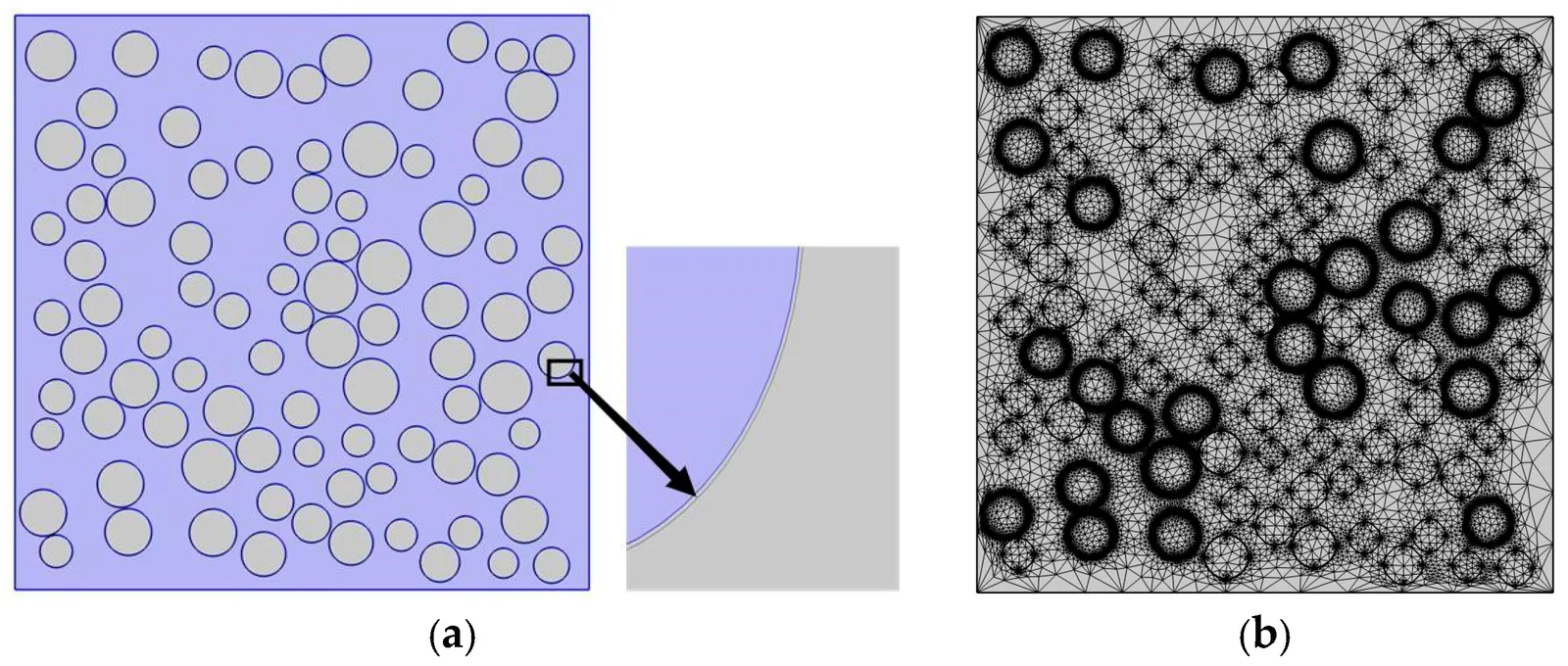
Two-dimensional concrete model incorporating the ITZ: (**a**) 2D model incorporating the ITZ; (**b**) Mesh of the 2D model incorporating the ITZ.

A two-dimensional mesoscopic model with an aggregate volume fraction of 34.4% was established. Aggregates were randomly distributed within the mortar matrix, and an ITZ with a thickness of 50 μm and a pore area fraction of 24% was placed around each aggregate. The two-dimensional model incorporating the ITZ is shown in Figure 8.

**Figure 9:**
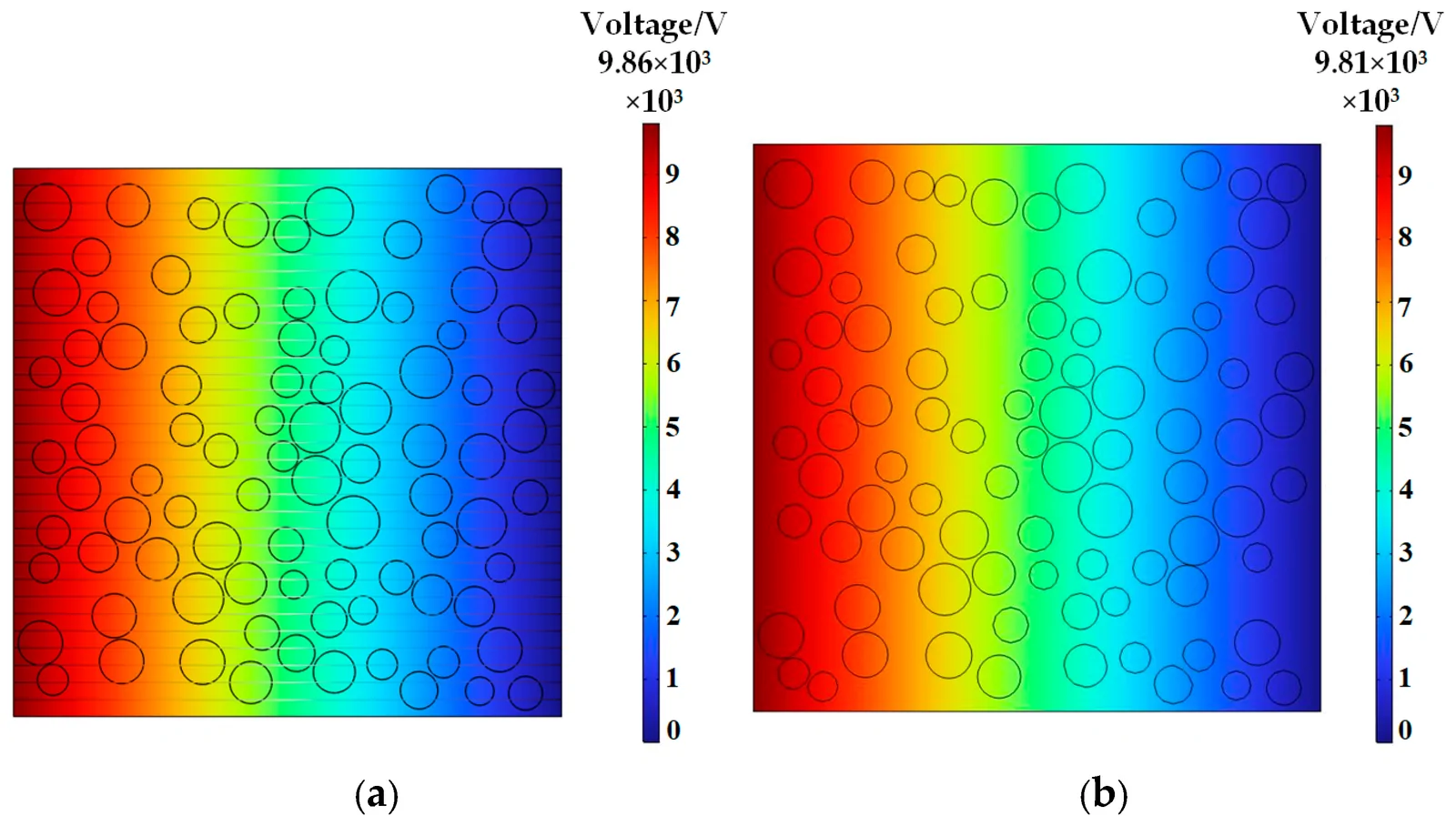
Two-dimensional electric potential distributions in concrete: (**a**) with ITZ; (**b**) without ITZ.

The electric current field in the concrete models with and without an ITZ was solved using the governing equations and boundary conditions described in Section 2.3. The resulting electric potential distributions are shown in Figure 9.

As shown in Figure 9, the calculated electric potential distributions of the models with and without the ITZ were very similar. Their calculated resistivities were 986 Ω·m and 981 Ω·m, respectively, corresponding to a relative difference of approximately 0.5%. Under the fixed electrical properties and model assumptions adopted in this study, this result indicates that the macroscopic equivalent electrical resistivity was not sensitive to the ITZ. This limited sensitivity is attributed to the small thickness and volume fraction of the ITZ relative to the aggregate and mortar phases. Consequently, the ITZ did not form a continuous pathway that dominated the overall electrical response. Moreover, current conduction was governed primarily by pore-solution connectivity and ion mobility within the mortar matrix, whereas the aggregates and their surrounding transition zones caused only local disturbances in the current paths [35]. Li et al. [19] similarly distinguished between the electrical and mechanical roles of the ITZ, reporting a limited influence on equivalent electrical resistivity while recognizing its importance in crack initiation and damage propagation. Based on the present electrical sensitivity analysis, the ITZ was omitted from the subsequent three-dimensional simulations, and a two-phase mortar–aggregate model was adopted to reduce geometric and computational complexity.

However, the approximately 0.5% difference reflects only the influence of the assigned ITZ electrical resistivity on the macroscopic equivalent resistivity and does not demonstrate that the ITZ is physically insignificant under subzero conditions. The sensitivity analysis assigned a fixed electrical resistivity to the ITZ and did not consider changes caused by local pore-water freezing, ice formation, or microcracking. The ITZ has a relatively loose and porous microstructure [26]. Owing to bleeding, water accumulates around coarse aggregates, particularly near their lower surfaces. Under subzero conditions, phase transformation of the accumulated water generates relatively high frost-heaving stresses in these water-rich interfacial regions, causing local interface damage or aggregate–mortar debonding [31]. Freezing stress also promotes the initiation, propagation, and eventual interconnection of microcracks within concrete [2]. Omitting the ITZ therefore removes the spatial region in which freezing-induced deterioration and damage localization develop around coarse aggregates. In addition, because the present model includes only the Electric Currents interface and does not couple heat transfer, pore-water phase transformation, or mechanical damage, it cannot reproduce ice crystallization pressure, crack initiation, or damage-induced changes in electrical properties. This simplification reduces the physical completeness of the model for describing freezing-induced damage. Therefore, the resulting two-phase model represents only the equivalent electrical conduction response and does not describe the mechanisms of freezing-induced structural degradation.

### 3.4. Effect of CLFP Dosage on Mortar Resistivity and Parameter Fitting

As demonstrated in Section 3.3, the ITZ had little influence on the overall resistivity of the concrete. A two-phase mortar-matrix–aggregate model was therefore used in the subsequent simulations. In this model, the mortar matrix represents the continuous conductive phase, and changes in its resistivity at subzero temperature directly govern the variation in the effective resistivity of the concrete.

**Figure 10:**
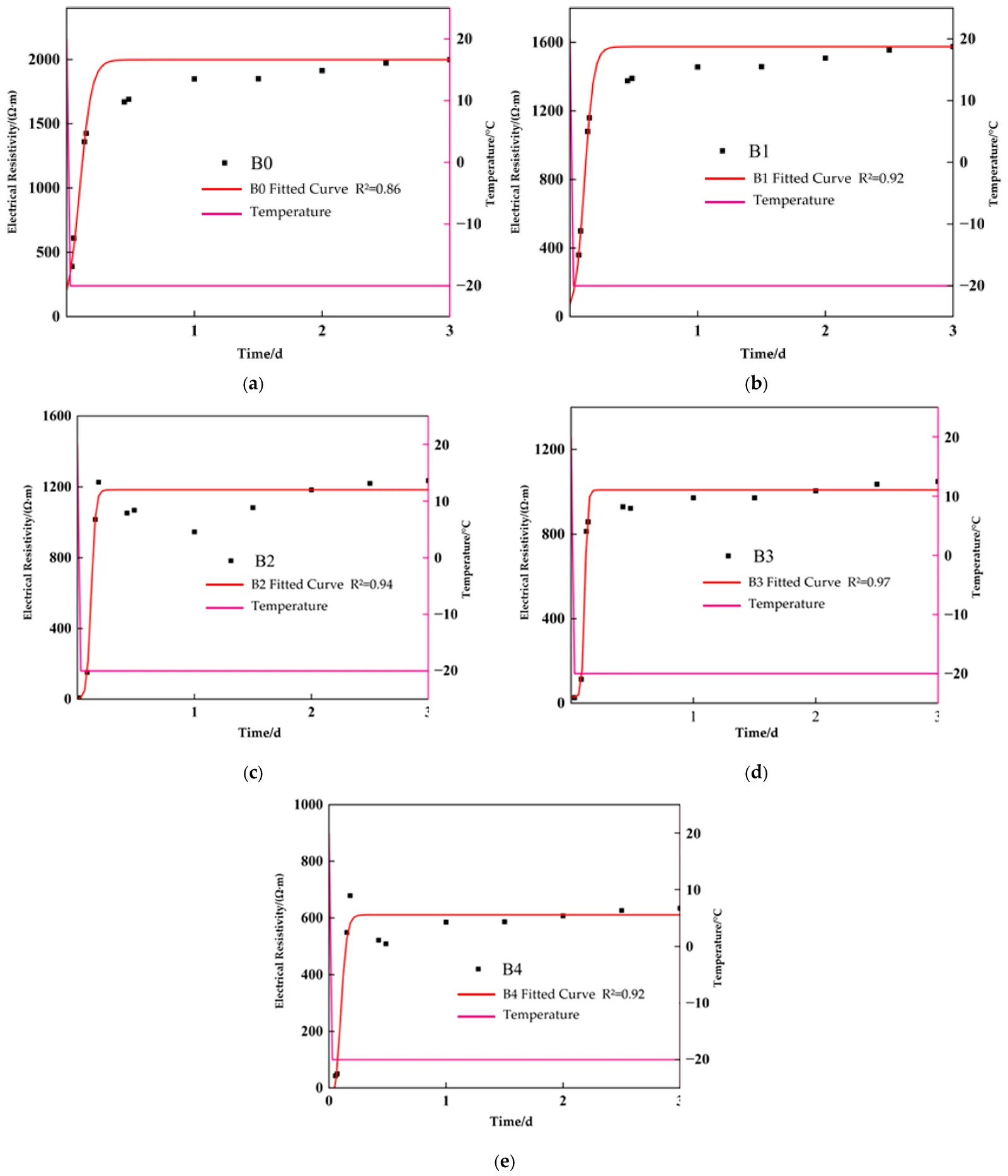
Electrical resistivity and fitted parameter curves of mortars with different CLFP dosages at subzero temperatures: (**a**) fitted subzero temperature resistivity curve of B0; (**b**) fitted subzero temperature resistivity curve of B1; (**c**) fitted subzero temperature resistivity curve of B2; (**d**) fitted subzero temperature resistivity curve of B3; (**e**) fitted subzero temperature resistivity curve of B4.

To determine the resistivity of the mortar matrix at different CLFP dosages, resistivity tests were conducted on mortar mixtures B0–B4 at −20 °C. Functions were fitted to the experimental curves, and the fitted results were used as material parameters in the COMSOL electric current field simulations, as shown in Figure 10.

As shown in Figure 10a–e, the resistivity of each mortar mixture during exposure to a constant temperature of −20 °C initially increased slowly, then increased rapidly, and finally approached a stable value, producing a distinct S-shaped curve. These changes corresponded to three stages: cooling and ice nucleation, rapid freezing, and stabilized freezing. During the initial subzero temperature stage, the decrease in mortar temperature increased the viscosity of the pore solution and reduced ion mobility, resulting in a gradual increase in resistivity. Ice crystals then formed rapidly and interrupted the liquid conductive pathways, causing a sharp increase in resistivity. After most of the freezable water had undergone a phase transition, the resistivity gradually stabilized [17,20].

As the CLFP dosage increased, the stabilized resistivity of the mortar generally decreased. This result indicates that CLFP increased the ionic concentration of the pore solution and reduced the extent of freezing, thereby preserving some liquid conductive pathways [27]. The S-shaped resistivity evolution was described using a Boltzmann function, with coefficients of determination, R^2^, exceeding 0.86 for all mixtures. The fitted Boltzmann equation is expressed as follows:

(6) $$\rho =\rho_{\infty }+\frac{\rho_{0}-\rho_{\infty }}{1+e^{\left[(t-t_{0})/\tau \right]}}$$

where *ρ* is the electrical resistivity of the mortar (Ω·m), *t* is the duration of subzero exposure (d), *ρ*_0_ is the estimated initial resistivity represented by the lower asymptote (Ω·m), *ρ*_∞_ is the stabilized resistivity represented by the upper asymptote (Ω·m), *t*_0_ is the time corresponding to the inflection point of the curve (d), and *τ* is the characteristic fitting parameter describing the steepness of the resistivity increase.

### 3.5. Experimental Validation and Simulation Results for Concrete with Different CLFP Dosages

**Figure 11:**
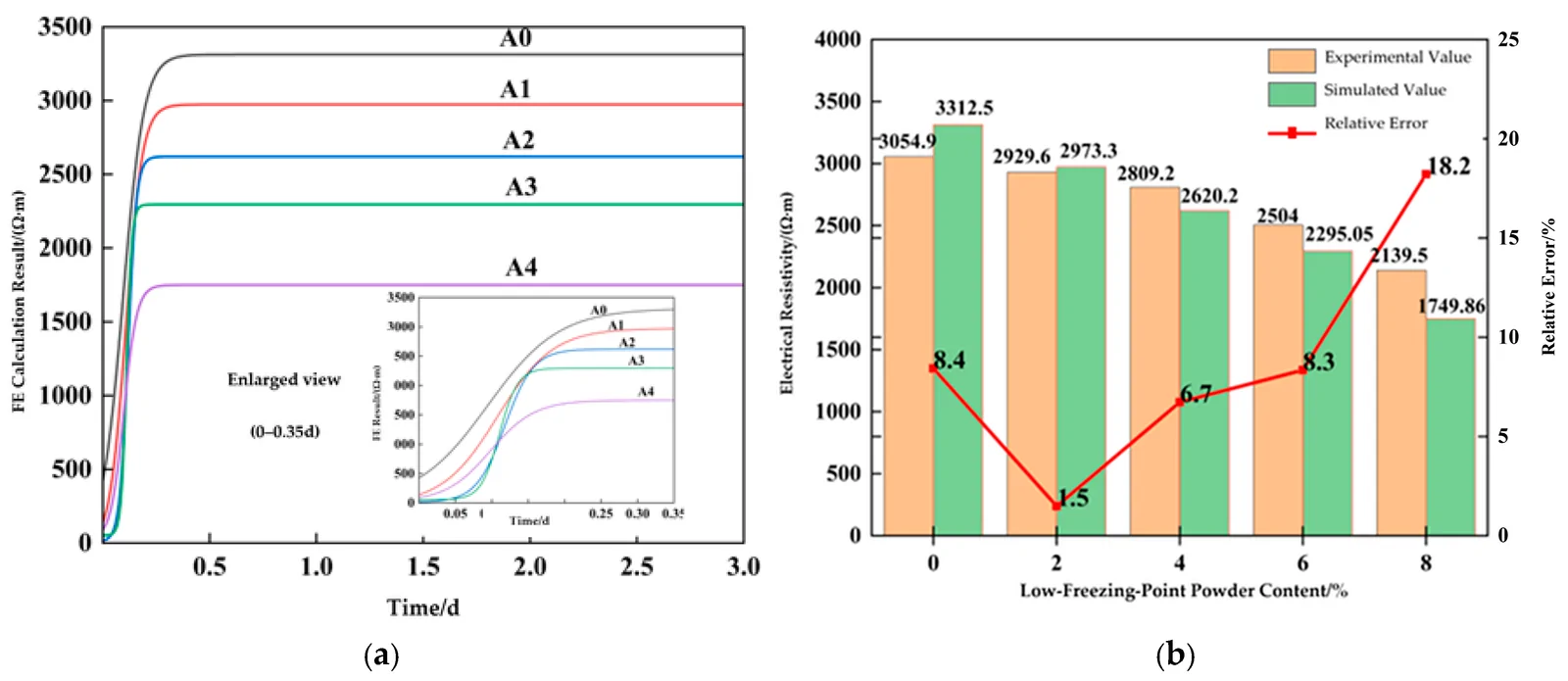
Comparison of the experimental and simulated electrical resistivity of concrete with different CLFP dosages: (**a**) simulated resistivity curves of concrete with different CLFP dosages; (**b**) experimental and simulated resistivity of concrete with different CLFP dosages.

The conductivity functions fitted in Section 3.4 were assigned to the corresponding concrete models A0–A4. Electric current field calculations were then performed to obtain the simulated resistivity of each concrete mixture. The simulation results were compared with the experimental measurements, as shown in Figure 11.

As shown in Figure 11a, after the conductivity functions of the mortar matrix at −20 °C were incorporated into the models, the effective resistivity of each concrete mixture exhibited an increasing curve similar to that measured experimentally. This result indicates that the time-dependent electrical properties of the mortar phase can be transferred to the overall resistivity response of concrete through the mesoscopic electric current field.

In the simulations, current was transmitted primarily through the continuous mortar phase. Because of their relatively high resistivity, the aggregates diverted and obstructed local current pathways, causing the effective resistivity of the model to exceed that of the mortar matrix alone. As the duration of subzero exposure increased, the prescribed mortar conductivity decreased, increasing the resistance of the existing conductive paths. With the aggregate geometry held fixed, this change caused the effective concrete resistivity to increase and gradually stabilize. These results illustrate how the prescribed mortar conductivity and aggregate obstruction jointly govern the simulated electrical response under subzero conditions. Tong et al. [36] reconstructed a mesoscopic model using CT data and validated the simulations against mechanical test results, providing a reference for the experimental validation of concrete mesoscopic models.

As shown in Figure 11b, the experimental and simulated stabilized resistivities exhibited generally consistent trends. As the CLFP dosage increased from 0% to 8%, the measured resistivity decreased from 3054.9 Ω·m to 2139.5 Ω·m, corresponding to a reduction of 29.96%, whereas the simulated resistivity decreased from 3312.5 Ω·m to 1749.86 Ω·m, corresponding to a reduction of 47.17%. Both datasets therefore showed a decrease in concrete resistivity with increasing CLFP dosage. The mesoscopic model using measured mortar conductivity as input reproduced this overall dosage-dependent trend. The relative errors between the simulated and measured stabilized resistivities for A0–A4 were 8.4%, 1.5%, 6.7%, 8.3%, and 18.2%, respectively, with an average relative error of 8.6%. The relative errors for A0–A3 were below 10%, indicating comparatively close agreement in stabilized resistivity. For A4, the relative error reached 18.2%, with the simulated value below the measured value, indicating that the model overestimated the reduction in resistivity at a CLFP dosage of 8%.

As shown in Table 5, A1 exhibited the closest agreement between the simulated and measured initial resistivities, whereas larger discrepancies occurred in the other mixtures. The fitted characteristic time decreased from 0.0488 d for A0 to 0.0126 d for A3 and then increased to 0.0304 d for A4. An approximate quantitative comparison over the complete 0–3 d interval, using the experimental records and simulation curves reconstructed from the figure, yielded time-weighted root mean square errors (RMSEs) of 429.85 Ω·m, 253.64 Ω·m, 252.75 Ω·m, 319.45 Ω·m, and 405.94 Ω·m for A0–A4, respectively. After normalization by the measured resistivity range of each mixture, the corresponding errors were 14.68%, 9.02%, 9.37%, 12.88%, and 19.23%. A1 and A2 showed comparatively closer agreement over the complete interval. For all mixtures, the RMSE during 0–0.35 d exceeded that during 0.35–3 d, indicating larger discrepancies in the early resistivity evolution. A4 exhibited the largest normalized full-curve error and the largest late-stage RMSE, at 372.08 Ω·m, demonstrating discrepancies in both the rising and stabilized stages.

The agreement obtained here should be interpreted within the scope of the prescribed mortar conductivity inputs; predictions for untested CLFP dosages or curing conditions require the corresponding mortar conductivity functions to be established first. The relative errors below 10% for the stabilized resistivity of the 0–6%CLFP mixtures support the use of the decoupled electrical model for this specific response under the tested conditions. This agreement does not validate its ability to describe freezing-induced mechanical deterioration. Evaluation of frost damage and strength retention requires additional mechanical and damage measurements and explicit representation of the relevant coupled processes.

Overall, the model captured the increase and subsequent stabilization of concrete resistivity under subzero conditions and provided relatively good agreement with the measured stabilized resistivity for CLFP dosages of 0–6%. It also reproduced the decrease in stabilized resistivity with increasing CLFP dosage. However, agreement at the stabilized stage did not imply equivalent agreement over the complete time history. The larger discrepancies during the early rising stage and for the 8%CLFP mixture indicate that further refinement is needed to describe the transient electrical response and the behavior at high dosages.

## 4. Conclusions

This study investigated the effects of calcium nitrate–sodium nitrite composite low-freezing-point powder (CLFP) content on the electrical resistivity and compressive strength of concrete through curing at −20 °C followed by positive-temperature curing. A random-aggregate mesoscopic model was also developed to analyze the current conduction characteristics of concrete. The main conclusions are as follows:

(1) During curing at −20 °C, the electrical resistivity of all concrete specimens exhibited three stages: a slow increase, a rapid rise, and stabilization. As the CLFP content increased from 0% to 8%, the resistivity-derived apparent freezing onset time increased from 4.32 h to 4.75 h, while the stabilized electrical resistivity decreased by 29.96%. These results indicate that CLFP delayed the onset of the rapid resistivity-rise stage and altered the stabilized electrical conduction state. The identified characteristic time was used to compare the freezing-related electrical responses of mixtures with different CLFP dosages.

(2) CLFP dosage affected both the resistivity-derived apparent freezing onset time and compressive strength development. Among the mixtures tested, the 2% CLFP mixture achieved the highest compressive strength, whereas the 8% CLFP mixture exhibited the greatest delay in apparent freezing onset. After 28 d of positive-temperature curing, the compressive strengths of the 2% and 4%CLFP mixtures were approximately 7.1% and 0.9% higher than that of the reference mixture, respectively, whereas those of the 6% and 8%CLFP mixtures were approximately 31.3% and 27.9% lower. The 4%CLFP mixture maintained compressive strength close to that of the reference mixture while exhibiting a later apparent freezing onset. Based on these two measured indicators, 4%CLFP was considered a suitable dosage under the present experimental conditions.

(3) The electrical resistivity of concrete was mainly governed by the electrical properties of the mortar phase and the blocking effect of aggregates. Under the adopted model parameters, including the ITZ changed the equivalent resistivity by approximately 0.5%, indicating a limited influence. The mortar–aggregate two-phase model reproduced the stabilized resistivity of mixtures containing 0–6%CLFP with relative errors below 10%, whereas the relative error reached 18.2% at 8%CLFP. Full-curve comparisons revealed larger discrepancies during the early rising stage, with A1 and A2 showing comparatively closer agreement over the complete time interval. Therefore, the model provided a better description of stabilized resistivity than of the early transient response, and further refinement is needed to simulate resistivity evolution at high CLFP dosages.

This study combined resistivity monitoring, compressive strength testing, and mesoscopic electrical modeling to evaluate the effects of CLFP under −20 °C exposure for 3 d followed by above-zero curing. The findings apply to the mixtures and curing regime investigated. The interpretations of hydration and microstructural mechanisms were informed by published studies, as microstructural changes and freezing-induced damage were not directly measured. Further research will extend the investigation to different subzero temperatures, exposure durations, and repeated freeze–thaw cycles, incorporating microstructural characterization and direct damage assessment. Coupled thermal, mechanical, damage, and electrical modeling will further clarify the relationship between resistivity evolution and frost damage.

## Figures and Tables

**Table 1:** Mix proportions of the concrete mixtures.

Group	Water/kg/m^3^	Cement/kg/m^3^	Sand/kg/m^3^	Coarse Aggregate/kg/m^3^	Water-Reducing Admixture/kg/m^3^	Calcium Nitrate Tetrahydrate/kg/m^3^	Sodium Nitrite/kg/m^3^
A0	240	500	870	930	5	0	0
A1	240	500	870	930	5	7.5	2.5
A2	240	500	870	930	5	15	5
A3	240	500	870	930	5	22.5	7.5
A4	240	500	870	930	5	30	10

**Table 2:** Principal physicochemical properties of calcium nitrate tetrahydrate and sodium nitrite.

Chemical Formula	Molar Mass/g·mol^−1^	Melting Point/°C	Density/g·cm^−3^	Water Solubility	Appearance
Ca(NO_3_)_2_·4H_2_O	236.15	44	1.86	Readily soluble	Colorless or white crystalline solid
NaNO_2_	69.00	271	2.168	Readily soluble	White or slightly yellow crystalline solid

**Table 3:** Effect of CLFP dosage on the characteristic values of concrete subjected to freezing.

Mixture	Initial Resistivity/(Ω·m)	Stabilized Resistivity After 3 d of Subzero Curing/(Ω·m)	Apparent Freezing Onset Time/d	Freezing Stabilization Time/d	Fitted Characteristic Time/d
A0	126	3054.9	0.180	0.263	0.028
A1	118.5	2929.6	0.183	0.288	0.036
A2	111	2809.2	0.185	0.254	0.024
A3	24	2504	0.194	0.217	0.008
A4	29	2139.5	0.198	0.212	0.005

**Table 4:** Mean values and variability of the 28 d compressive strength for different CLFP dosages.

Mixture	Number of Specimens	Mean Compressive Strength/MPa	SD/MPa	CV/%
A0	3	35.1	0.361	1.03
A1	3	37.6	0.458	1.22
A2	3	35.4	0.608	1.72
A3	3	24.1	0.361	1.50
A4	3	25.3	0.436	1.72

**Table 5:** Characteristic values of simulated resistivity curves at different CLFP dosages.

Mixture	Initial Resistivity/(Ω·m)	Stabilized Resistivity After 3 d of Subzero Curing/(Ω·m)	Apparent Freezing Onset Time/d	Freezing Stabilization Time/d	Fitted Characteristic Time/d
A0	425.59	3312.50	0.0170	0.2451	0.0488
A1	139.52	2973.30	0.0255	0.2222	0.0380
A2	16.50	2620.20	0.0612	0.1758	0.0196
A3	52.49	2295.05	0.0734	0.1476	0.0126
A4	90.39	1749.86	0.0263	0.1888	0.0304

## Data Availability

The data presented in this study are available from the corresponding author upon reasonable request.
